# A System for Simple Real-Time Anastomotic Failure Detection and Wireless Blood Flow Monitoring in the Lower Limbs

**DOI:** 10.1109/JTEHM.2016.2588504

**Published:** 2016-08-25

**Authors:** Michael A. Rothfuss, Nicholas G. Franconi, Jignesh V. Unadkat, Michael L. Gimbel, Alexander STAR, Marlin H. Mickle, Ervin Sejdić

**Affiliations:** Department of Electrical and Computer EngineeringSwanson School of EngineeringUniversity of PittsburghPittsburghPA15261USA; Department of Plastic SurgeryUniversity of PittsburghPittsburghPA15261USA; Department of ChemistryUniversity of PittsburghPittsburghPA15260USA

**Keywords:** Anastomosis, bedside monitor, blood flow monitor, continuous wave, Doppler, flowmeter, free flap

## Abstract

Current totally implantable wireless blood flow monitors are large and cannot operate alongside nearby monitors. To alleviate the problems with the current monitors, we developed a system to monitor blood flow wirelessly, with a simple and easily interpretable real-time output. To the best of our knowledge, the implanted electronics are the smallest in reported literature, which reduces bio-burden. Calibration was performed across realistic physiological flow ranges using a syringe pump. The device’s sensors connected directly to the bilateral femoral veins of swine. For each 1 min, blood flow was monitored, then, an occlusion was introduced, and then, the occlusion was removed to resume flow. Each vein of four pigs was monitored four times, totaling 32 data collections. The implant measured 1.70 cm^3^ without battery/encapsulation. Across its calibrated range, including equipment tolerances, the relative error is less than ±5% above 8 mL/min and between −0.8% and +1.2% at its largest calibrated flow rate, which to the best of our knowledge is the lowest reported in the literature across the measured calibration range. The average standard deviation of the flow waveform amplitude was three times greater than that of no-flow. Establishing the relative amplitude for the flow and no-flow waveforms was found necessary, particularly for noise modulated Doppler signals. Its size and accuracy, compared with other microcontroller-equipped totally implantable monitors, make it a good candidate for future tether-free free flap monitoring studies.

## Graphical abstract

Current totally implantable wireless blood flow monitors are large and cannot operate nearby other blood flow monitors. The developed system monitors blood flow wirelessly, with a simple/easily interpretable real-time output. The system was calibrated across expected venous flow rates using a syringe pump. Eight measurements were taken from the bilateral femoral veins in each hind leg of four pigs. The implant measured 1.70 cm3 without battery/encapsulation, the smallest reported in literature, and the relative error was between -0.8% to +1.2% at its largest calibrated flow rate. After establishing the relative flow and no-flow waveform amplitudes, flow interpretation is simple.

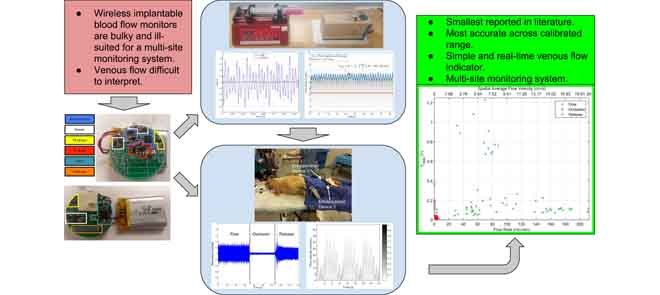

## I. Introduction

Microvascular free tissue transfer (i.e., free flap) refers to a class of procedures used for reconstructing anatomic defects [1]–[4], often due to cancer treatment [5], [6], infection, or trauma [7], [8]. These procedures involve the transfer of a block of tissue (i.e., flap) from a donor site (e.g., abdomen, leg) to reconstruct a major defect in another region of the body (e.g., breast, mandible) [9], [10]. Different from simple skin grafting [11], this transfer requires microsurgical connections (anastomoses [12]–[14]) of veins and arteries to be established between the flap and the new site [15]. Unfortunately, blood vessel patency (i.e., openness of a vessel) can be compromised in up to 10% of cases in the first several days after surgery [16], [17]. This is largely due to vessel thrombosis, compression, kinking, or tension, which creates a surgical emergency, because the transferred flap will fail unless blood flow is reestablished promptly [18]–[20]. Flap failure results in increased cost [21], patient morbidity [22], [23], and even death [11], [24]. As a result, reconstructive plastic surgeons maintain a low threshold for returning to the operating room to investigate a suspected vascular problem, resulting in an undesired, but accepted, risk of negative (i.e., preventable) re-exploration (i.e., up to 30% in head and neck free flaps [25]) [26], [27]. However, these unnecessary surgical re-explorations are also associated with increased morbidity and expense (up to $20,000-$30,000/event [28]).

In order to help detect loss of blood flow expeditiously [29], [30], indirect [31]–[35] and direct flow detection devices [36]–[38] are used [39]. The current gold standard for monitoring of free flap surgeries is a wired Doppler device in which a piezoelectric transducer crystal sensor is loosely attached to a silicon cuff that encircles the monitored vessel [40]–[43]. The sensor is designed to easily separate from the silicon cuff so that when the critical monitoring period (i.e., 4 to 7 days) is complete, the wire and sensor can simply be pulled out, leaving the silicon cuff in place. The sensor generates an insonating ultrasonic signal and receives the weak, backscattered signal from flowing red blood cells [44]–[46]. Loss of the signal may indicate loss of blood flow, but may also result from accidental internal separation of the sensor from the cuff, thereby creating a false positive [27], [47]. Thus, the purposeful design of the wired Doppler that allows wire/sensor removal also creates an inherently high risk of false positive alerts. Occasionally, the sensor may be too adherent to the silicon cuff, such that wire withdrawal creates a kink or injury to the vessel [17]. It stands to reason that a novel, wireless Doppler monitoring device will eliminate the deficiencies of the existing gold standard system by omitting the main source of problems, the wire. Such a wireless device would be totally implantable and remain implanted, rather than removed as in the case of the gold standard. In summation, direct devices suffer from several shortcomings, including: recognized accidental probe dislodgement which makes monitoring impossible without further surgery, up to 30% false positive rate due to unrecognized internal probe dislodgement, leading to unnecessary surgery, risk of injury to the blood vessels upon probe withdrawal, complex blood flow signals requiring expert (i.e., rather than bedside nurse) interpretation, and these devices are also cumbersome.

The majority of reported implantable wireless Doppler blood flow monitors rely primarily on custom analog electronics and/or accompanying digital control circuitry (e.g., [48]–[52]). However, recent implantable wireless Doppler blood flow monitor developments have reported devices which incorporated microcontrollers [53]–[55]. The microcontrollers, which are comprised of a microprocessor and additional peripheral functions (i.e., analog-to-digital converters, serial communication devices, controllable digital input and output ports, etc.), provide a platform for software customization and controllability of a system. Customizable software can leverage devices that can be dynamically modified to satisfy an application.

We have developed a prototype wireless implantable blood flow monitoring device to solve the problems associated with the wired Doppler device in free flap monitoring. In free flap monitoring, the venous outflow is typically monitored, because it also indicates arterial inflow to the flap. However, monitoring venous blood flow, particularly in the lower limbs, is especially difficult, because its detection hinges on the experience of available personnel [56], [57]. Our device targets the venous outflow of free flaps, and in particular, the case where anastomotic failure occurring in the lower limbs. Prior work in the field has not focused on easing interpretation of the flow information, which is expected to reduce the demand for experienced ultrasound operators for this task.

Previous devices incorporating microcontroller units have not addressed reducing size through noise and interference management as well as incorporating highly integrated electronics and components. By addressing size in this manner, we have achieved the smallest device footprint utilizing a microcontroller unit (i.e., electronics volume, including antenna, is about 1.7 cm^3^). Additionally, we have developed these devices as part of a system, to solve the problem of actuating and operating a single device when multiple devices are nearby, which has also not been addressed in any literature to date.

## II. System Description and Methods

### A. Implanted Transmitter

#### 1) Continuous Wave Doppler Configuration

**FIGURE 1.**
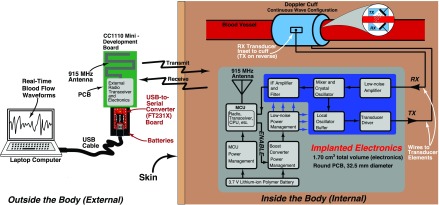
Block diagram of the implemented wireless Doppler blood flow monitoring system.

Figure 1 shows the implemented Doppler system used in this research. The continuous wave (CW) Doppler implementation has two piezoelectric transducers, one for transmitting (TX) ultrasonic energy, and one for receiving (RX) scattered ultrasonic energy. An electrical signal excites the transmitting transducer, which converts the electrical energy to mechanical energy. Mechanically pushing the face of the piezoelectric material produces a longitudinal wave that propagates away from the transducer face. Once the ultrasonic wave launches from the transmitting transducer, the wave scatters on objects, such as red blood cells, at a frequency deviation (i.e., from the frequency of the impinging wave) proportional to the velocity of the scattering elements. The scattered energy is collected and transduced (i.e., the mechanical wave pushes the piezoelectric transducer face to produce an electrical signal) by the receiving transducer. This effect is described by the well-known Doppler Equation (i.e., $f_{d} = \frac {2 v f_{0}\cos \theta }{c}$), where $f_{d}$ is the frequency deviation from the impinging ultrasonic frequency, $f_{0}$, $v$ is the velocity of the moving scatterers insonated by the impinging ultrasonic beam, $\theta $ is the angle between a vector normal to the transducer’s face and the axis along the direction of blood flow, and $c$ is the speed of the ultrasonic wave in the specific media (e.g., blood). Further detail and a full treatment of Doppler ultrasound physics can be found in Shung as well as Boote [46], [58].

The transducer apparatus holds the two transducers in a CW configuration, inset to a cuff, custom manufactured by Iowa Doppler Products (Iowa City, IA). The cuff was designed for a vessel with an outer diameter of 5 mm, and the cuff itself was designed to be semi-rigid so as to prevent misalignment of the transducers (i.e., modifying the sample volume). The transducers were a 1 mm diameter piezoelectric material, manufactured to operate at 20 MHz, with a transducer angle, $\theta $, of 45 degrees.

#### 2) Implanted Hardware and Software

##### a: Implanted Electronics

The Doppler electronics are a unidirectional configuration (i.e., blood flow direction cannot be detected), as opposed to bidirectional (i.e., blood flow direction can be detected), in order to reduce the complexity and size of the implant. For laminar flow applications, such as in this study, a unidirectional configuration is appropriate.

**FIGURE 2.**
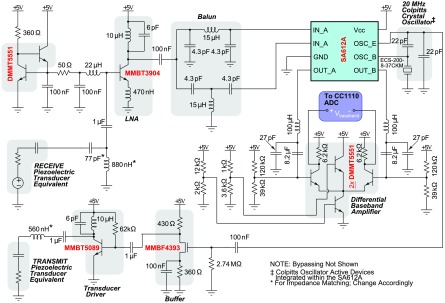
Schematic of the implanted Doppler electronics, excluding power management and the MCU.

The electronic hardware architecture for a continuous wave Doppler is comprised of several core elements. The circuit of Figure 2 shows the analog circuitry used to excite the transmitting transducer and receive and demodulate Doppler blood flow signals. Starting from the transducer driver, a quartz crystal-based Colpitts oscillator (i.e., active device internal to the SA612A, by NXP Semiconductors, Eindhoven, Netherlands) provides a carrier frequency reference, $f_{0}$ in the Doppler equation. The transducer driver and mixer local oscillator (LO) must use the same frequency reference, otherwise inevitable frequency drifts between the two will result in in-band baseband components. The oscillator reference is buffered and amplified to provide sufficient drive for the transmit transducer.

Backscattered signals transducer by receiving transducer drive the input of the low-noise amplifier front-end. The LNA is biased with a simple current mirror from a matched pair of bipolar transistors. The LNA is typical a common-source configuration, which used the MMBT3904 (Fairchild Semiconductor International, Inc., San Jose, CA) bipolar transistor to achieve a low noise figure (i.e., about 3–4) for the expected range of source resistance (i.e., the real component of the transducer’s impedance when the reactive component is tuned out). The LNA’s load is formed by the LC tank, tuned to $f_{0} = 20 MHz$, and the lumped element balun’s single-ended input impedance. The balun converts the single-ended amplified signal to a differential signal for the mixer’s (SA612A) RF input ports. Additionally, the balun impedance-matches the mixer’s RF input port impedances to a lower single-ended impedance to load the LNA. The LO is generated by the crystal-based Colpitts oscillator of the SA612A. It should be noted that the double-balanced mixer operates as part of a homodyne receiver signal chain. Any frequency deviation from the LO ($f_{0}$) results in baseband conversion about zero-frequency. The intermediate frequency (IF) output of the mixer is taken differentially, low-pass filtered, and then amplified differentially before driving the differential inputs of a microcontroller unit’s (MCU) analog-to-digital converter (ADC).

**FIGURE 3.**
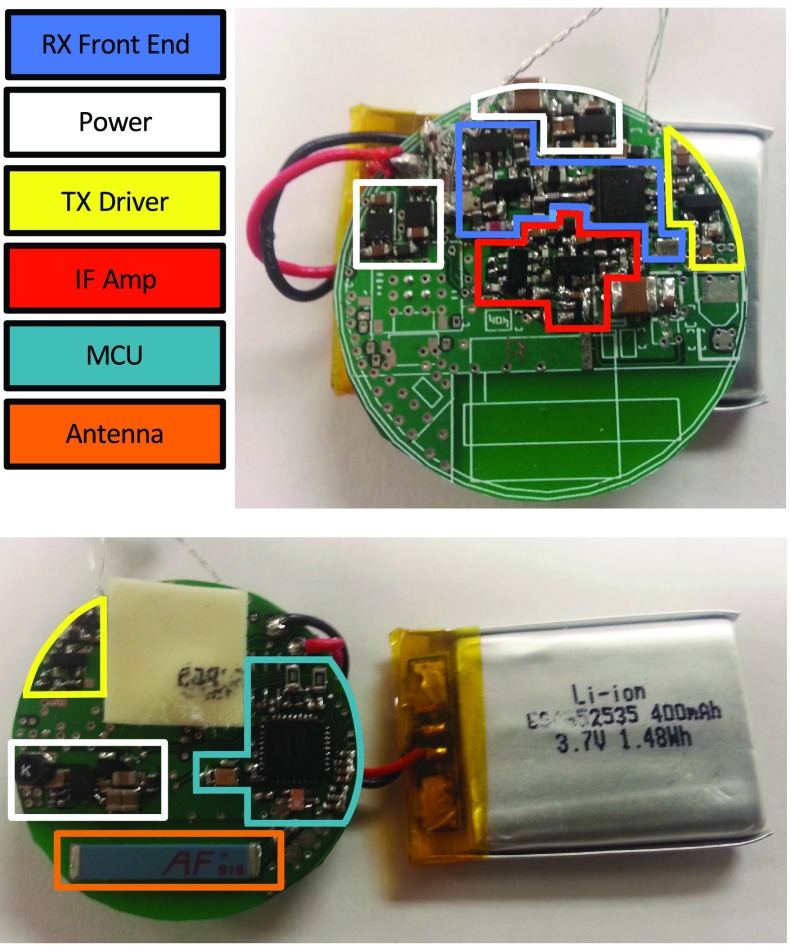
Hardware components labeled on the implanted device PCB.

The MCU, CC1110F32 by Texas Instruments (Dallas, TX), provides many functions in a single package that minimize the circuit complexity and occupied PCB real-estate. The MCU’s on-board ADC was configured for 10-bit differential operation with the MCU’s internal 1.25 V reference, corresponding to 1024 quantization levels across a 2.5V range (i.e., ±1.25 V and a 2.441 mV resolution). The MCU’s radio can operate across several Industrial Scientific Medical (ISM) frequency bands (i.e., 315 MHz, 433 MHz, 868 MHz, and 915 MHz). While some ISM bands are subject to greater interference due to overcrowding, this problem can be reduced by the fact that the external receiver will be very near the implant location (i.e., <1 meter). For this research, 915 MHz was chosen. The primary metric for the presented device is minimizing its size. Higher frequencies permit smaller antennas than lower frequencies. Despite higher power losses in tissue for higher frequencies, the high receiver sensitivity of the CC1110F32 still provides a large communication link budget. Additionally, a chip balun, rather than a lumped element balun, saves space between the MCU’s differential RF ports and the single-ended antenna. An omni-directional 1/4-wave ceramic chip antenna (16.0 mm $\times3.0$ mm $\times1.7$ mm) in a surface mount package is used (MFR P/N: ANT-916-CHP-T, Linx Technologies Inc., Merlin, OR). The antenna’s usable bandwidth is 10 MHz, and it has a maximum gain of 0.5 dBi. The analog electronics (i.e., Figure 2) are powered through a low-noise low-dropout regulator, which is driven by a boost converter (i.e., to raise the battery cell voltage), which is enabled by the MCU to collect blood flow data, and disabled to save power otherwise. The device is labeled with the major components on its PCB in Figure 3.

A $100-1000 \mu \text{L}$ Fisherbrand Elite Adjustable-Volume Pipetter (ThermoFisher Scientific Inc., Waltham, MA) set to $100~\mu \text{L}$ increments was used to measure the volumetric water displacement of the device’s electronics (excluding the transducer and battery). The measurements were repeated five times and averaged.

##### b: Battery Life and Encapsulation

The choice of battery for the implantable blood flow monitor was a 3.7 V 400 mAh lithium-ion polymer battery from Great Power Battery Co. Ltd (Kowloon, Hong Kong). Its volume was 4.4 cm^3^ (5 mm $\times25$ mm $\times35$ mm), which fits nearly within the footprint of the implantable blood flow monitor, so as to not add unnecessary bulk. When the MCU is in the active mode (i.e., the implantable device is fully operational and sensing blood flow and transmitting wireless data), the implantable device continuously consumes about 120 mA from a 3.7 V power supply, which provides a theoretical maximum of 3 hours and 20 minutes of continuous run time from the battery. When the MCU is in the sleep mode, the implanted device can remain dormant for over three weeks. Sleep modes can be modified to deliver a much longer dormant lifetime if the intermittent wake up state is activated less frequently (i.e., $\gg ~33$ seconds).

The experimental protocol in this research required sacrificing the animals upon completion of the data collection (i.e., less than half a day). Therefore, encapsulation to ensure biocompatibility for a lengthy implant lifetime was not necessary, and thus, only a simple barrier to electrically protect the implant from tissue was used. To encapsulate the devices, the implant was first covered in generic blue painter’s tape followed by a coating with PlastiDip Synthetic Rubber Coating (Blaine, MN). The reason for the painter’s tape was to prevent adhesion of the PlastiDip coating to the electronics. Also, the painter’s tape facilitated easy encapsulation removal to salvage the devices after the experiments. To measure the fully encapsulated device (i.e., includes electronics, battery, and the encapsulation containing these), the same method was used as described in Section II-A2a but instead with the Adjustable-Volume Pipetter set for $500~\mu \text{L}$ increments.

##### c: Implanted Software State Machine and Multiple Device Arbitration

**FIGURE 4.**
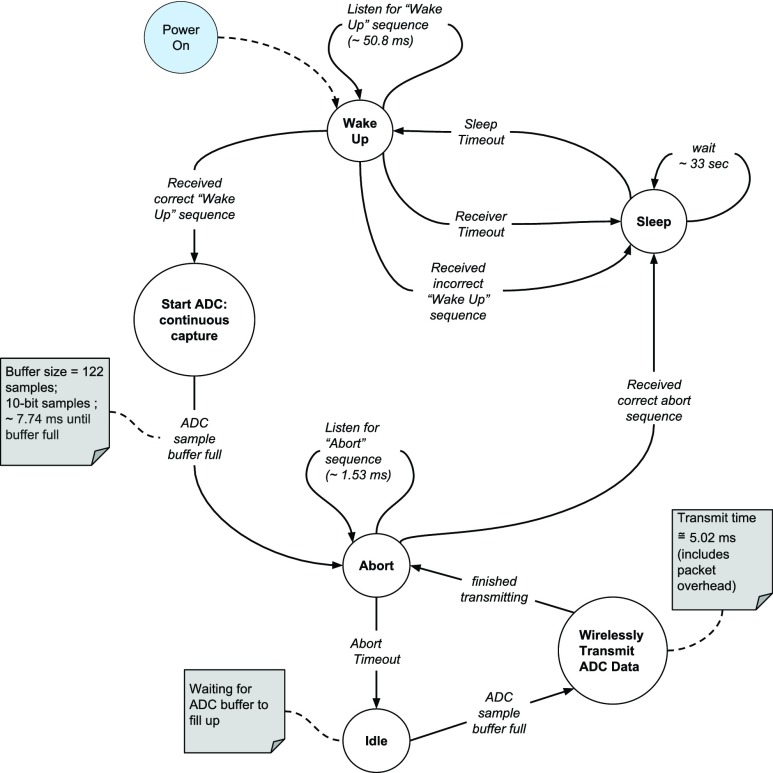
Software finite state machine on-board the implant.

The developed software finite state machine (FSM) (See Figure 4) on the implanted device arbitrates system functions (i.e., wireless communication, device activation, device abort, and blood flow waveform capture) and power modes (i.e., sleep and active). Additionally, a unique serial identifier (SID) is assigned to each device for targeted device activation, preventing interference from unintentionally activated devices and unnecessarily draining their batteries.

### B. External Hardware and Software

#### 1) Transceiver, Multiple Device Arbitration, and Real-Time Display

The external radio transceiver activates and deactivates implants, receives wireless data from implants, and transmits digital serial data via a serial universal asynchronous receiver/transmitter (UART) to a computer for processing, display, and storage. The external radio transceiver is a development board from the CC1110F32 Mini-Development Kit 868/915 MHz by Texas Instruments (Dallas, TX). An FSM diagram detailing the external device’s software is omitted, because its design can be inferred from the internal device’s software.

The data collection protocol (See Section II-C) dictates monitoring blood flow in the femoral vein in both the right and left leg, which required two devices. Without the ability to activate a specific device, both devices would turn on and transmit simultaneously, resulting in interference and unnecessary battery drain for one of the devices. The development board simplifies multiple device arbitration. The breakout header pins on the development board, which route to input/output pins on the MCU, offer a convenient method to select a device. The external development board is outfitted with an FT231X (Future Technology Devices International Ltd., Glasgow, UK) Breakout board. The FT231X chip on the breakout board performs serial-to-USB conversion. A custom program on-board the computer provides a way to observe the digitized blood flow waveforms in real-time, similar to an oscilloscope display. The waveform display was imperative for ensuring proper placement of the cuff on the vessel under test in the operating room, and it provided a simple visual indicator of occlusion/flow.

### C. Data Collection Protocol

The *in vitro* testing is necessary to benchmark the device before performing animal experiments. A small length of translucent heat-shrink tubing served as a mock vessel, which is similar to others’ methods [49]. The heat-shrink tubing was firmly affixed onto the end of a syringe, and its outer diameter was measured at 210 mils (5.33 mm) and its wall thickness was 11.5 mils (0.29 mm). The heat-shrink tubing was inserted into the cuff, and any remaining space between the tube and the cuff walls was filled with generic ultrasonic gel. The syringe was filled with a pink-colored blood phantom, available by Blue Phantom (Sarasota, FL). The acoustic properties of the blood phantom match closely with that of human blood. The syringe was mounted in a syringe pump (i.e., model no. NE-1000 by New Era Pump Systems, Inc., Farmingdale, NY), which provided a constant flow rate (±1% error) that could be referenced for performance evaluation purposes. Maximum flow rate with the NE-1000 is limited by a syringe’s inner diameter; the largest syringe that we could obtain held 50 mL (i.e., corresponding to a flow rate of 34.15 mL/min). The device was benchmarked from 0.00 mL/min to 10.0 mL/min in 1.00 mL/min increments, and from 15.0 mL/min to the maximum flow rate in 5.00 mL/min increments. Using the relationship for average volumetric flow rate, $Q = \overline {\mathbf {v}}\cdot \mathbf {A}$, where $\overline {\mathbf {v}}$ is the spatial average velocity of blood through the cross-sectional area and $\mathbf {A}$ is the lumen cross-sectional area [59] (i.e., $\mathbf {A}$ of the heat-shrink tubing is 0.18 cm^2^), and the Doppler equation (i.e., with $\theta = 45^{\circ }$, $c = 1570$ m/s, $f_{c} = 20$ MHz). The corresponding spatial average blood flow velocities for the reference flow rates (e.g., 3.2 cm/s for 34.15 mL/min) are within the expected physiological mean velocities of veins in the legs [60], which are the measurement targets for this study.

In order to compute flow volume, we assumed uniform ultrasonic insonation of the lumen and a laminar unidirectional flow direction. To obtain the flow velocity, first, the short-time Fourier transform (STFT) was applied to the time-domain baseband flow signal. Second, the envelope of the resultant STFT is computed in the time-frequency domain. Time-variations of the spectral content were expected in the collected performance evaluation data due to the motor driving the syringe pump (i.e., similar observations are visible in Doppler spectrograms for real-blood flow waveforms). Hence, third, the envelope was smoothed with either a moving minimum or moving maximum filter to reduce the effect of peaking, whichever reduces the measurement error the most. And lastly, the mean of the smoothed envelope is taken as the maximum velocity, $v_{max}$, across the collection period. The flow estimate, $Q$, is evaluated noting that the spatial average velocity, $\overline {\mathbf {v}}$, is $\tfrac {v_{max}}{2}$ for laminar flow within the lumen [61].

**FIGURE 5.**
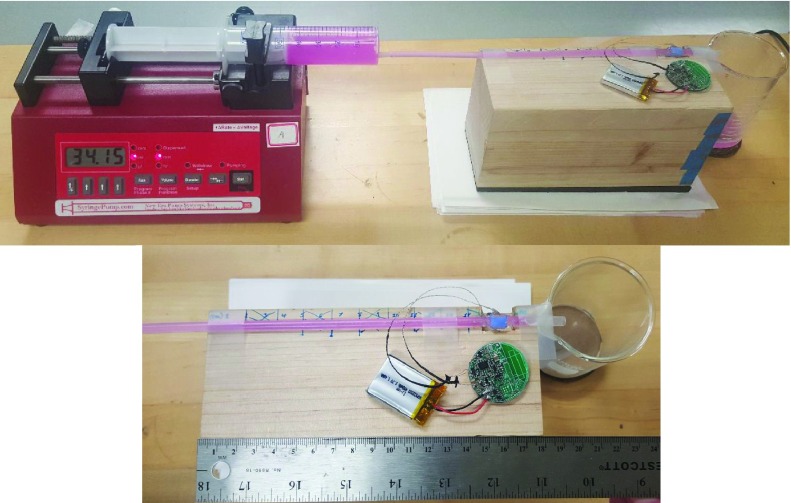
*In vitro* setup showing the Doppler device, syringe filled with the pink-colored blood phantom, and heat-shrink tube used as a mock blood vessel mounted in the syringe pump. Entrance length is about 15 cm.

An occlusive event (i.e., no flow) was simulated simply by disabling the syringe pump (i.e., the blood phantom and cuff remain in place, the same as with a flow event). The resulting waveforms for the tests were visible in real-time on the laptop screen. Figure 5 shows the setup for the *in vitro* testing. According to Fogel [62], an entrance length of 10 times the inner diameter of the tube is sufficient for 90% of the parabolic flow profile to develop. For the 0.47 cm tube used in the *in vitro* testing, the entrance must be at least 4.70 cm. In our set-up, we set the entrance length to approximately 15.0 cm. The tube was held straight using a groove inset to a wooden block.

The following experimental protocol, approved by the Institutional Animal Care and Use Committee (IACUC) at the University of Pittsburgh, was used for the *in vivo* testing of the wireless implantable Doppler blood flow monitoring devices. Four swine were prepared and anesthetized during the duration of the experiments and were sacrificed at the conclusion.

The human and swine vascular systems are comparable. The vascular anatomy, with regard to bifurcation patterns and sizes, are similar [63]. The size and concentration of red blood cells, which are the primary acoustic scatterers, for swine and humans are similar (i.e., $4- 8~\mu \text{m}$ at $5- 8\,\times \, 10^{6}/{mm^{3}}$ in pigs compared to $7.4- 9.4~\mu \text{m}$ at $4.6- 6.2\,\times \, 10{^{6}}/{mm^{3}}$ in human males) [46], making the animal model clinically relevant prior to human trials.

**FIGURE 6.**
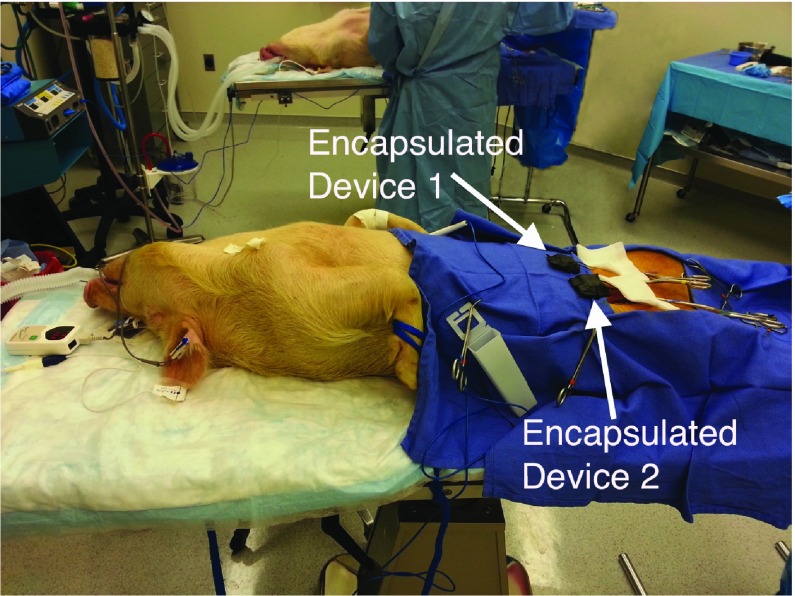
*In vivo* setup showing the Doppler devices connected to the bilateral femoral veins in both back legs of one pig. The encapsulated devices are shown exposed solely for photographic illustration.

The femoral vein was monitored in each of the back legs of the four swine, which was a good fit for the transducer cuff size; the blood flow analysis assumes the inner diameter to be the same as in our *in vitro* testing. To collect data from a pig, two wireless blood flow monitors were used, one for each leg. Ultrasonic gel was used to bridge air gaps between the piezoelectric transducer faces and the femoral vein during initial cuff placement around the vein. A suture was tightened around (i.e., a tourniquet) the femoral vein proximal to the transducer cuff to simulate an occlusion scenario (e.g., anastomotic failure in free flaps). First, blood flow was monitored for about one minute, followed by introducing the occlusive event (i.e., no-flow) and monitoring for about one minute, and last, the occlusion was removed and the resumed flow was monitored for about one minute. This protocol was repeated four times for each back leg for each animal. This resulted in 32 data collection cycles (i.e., eight collections from each swine with four collections for each back leg). The foreground in Figure 6 shows a pig with two Doppler devices, with each device affixed to one of the pig’s bilateral femoral veins in its back legs.

The device was implanted in a 1 cm-deep subcutaneous pocket of fat below the pig’s skin. Considering only the power loss (i.e., attenuation) due to material absorption, about 0.21 dB/cm may be lost in fat at 915 MHz [64]. Power loss due to skin is small and therefore neglected here, due to its relative thinness compared to other tissues. About 1.5 dB/cm power is lost in muscle at the same frequency. The receiver sensitivity of the external CC1110F32 at 500 kBaud at 915 MHz is -86 dBm, and the transmit power on the implant device was set to 10 dBm. The receiver was nominally about 1 meter away from the implant site, so the free space path loss would be about 32 dB. Therefore, provided the implant is not beneath muscle, about 64 dB remains in the link budget, which means that the amount of fat tissue between the implant and the receiver will not have a significant impact on the communication link.

If Specific Absorption Rate (SAR) limitations are imposed on the implant (i.e., the average power in 1 gram of tissue not to exceed 1.6 W/kg and the average power in 10 grams of tissue not to exceed 2 W/kg [65] over 6 minutes), the observation time, when the implant’s radio is transmitting, will be limited. Accurately determining SAR requires use of a microwave fields simulator (e.g., HFSS by ANSYS, Canonsburg, PA), because of the complexity of the tissues’ electromagnetic properties as well as their geometry (i.e., a skin-fat-muscle stack-up). However, a rough estimate of SAR shall be used in lieu of the development of a complex tissue and implant model.

In order to obtain a rough SAR estimate, the more stringent 1 gram SAR values, for antennas of a similar size (i.e., less than or equal to the size of this work’s PCB substrate, 1310 mm^3^, which includes the antenna ground counterpoise), are averaged (= 468.2 W/kg, for 1 W of delivered power to the antenna) from a 2012 literature review of implanted antennas for biomedical telemetry by Kiourti and Nikita [66]. A radio packet is transmitted every 7.74 ms; the packet transmission taking 5.02 ms, and the radio off-time is 2.72 ms (see Figure 4); all times were measured on a Saleae Logic 16 Logic Analyzer (South San Francisco, CA). The CC1110F32 is configured to deliver 10 mW to the implanted antenna; however, taking into account the duty cycle (64.9%) of wireless on-time due to packet transmission, the average power continuously delivered to the antenna is 6.49 mW. Using the maximum expected SAR of 468.2 W/kg from literature, and the maximum permissible SAR of 1.6 W/kg over 6 minutes as per regulation, the maximum allowable continuous power delivered to the antenna cannot exceed 3.4 mW ($=\frac {1.6 \:\: W/kg}{468.2 \:\: W/kg} * 1\:\: W$; calculated according to Kim and Rahmat-Samii [67]). Therefore, to meet this requirement, the observation period using the developed implant cannot exceed about 3 minutes and 9 seconds ($=\frac {3.4 \:\: mW}{6.49 \:\: mW}* 6$ minutes) for a single device during a 6 minute period. This estimate suggests that, for a single implanted Doppler flow monitor, the data collection protocol described earlier in this section complies with safety regulations for power absorbed by the human body.

## III. Results

The developed device was slightly larger than a U.S. Kennedy Half-dollar. The circular PCB substrate was about 32.5 mm in diameter and 1.58 mm (i.e., thick from top layer metal to bottom layer metal). The total implanted electronics volume, including the antenna but excluding the battery, was 1.70 cm^3^. The battery volume was 4.4 cm^3^, and the total encapsulated volume (excluding the transducer apparatus and leads) was about 18.0 cm^3^.

### A. In Vitro Performance

**FIGURE 8.**
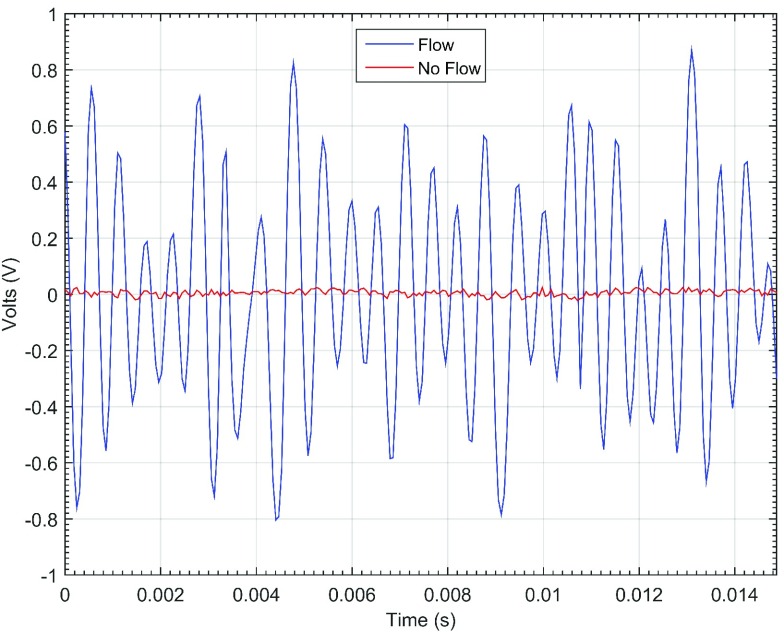
Oscilloscope-like view used on the laptop computer for real-time blood flow assessment. The displayed waveforms are the transduced baseband Doppler signals. Window length is 244 data samples long (i.e., 14.9 ms).

The *in vitro* testing served to characterize the performance of the device. Real-time waveform data was viewed on a laptop screen (Figure 8, reproduced in MATLAB; also shown are time and voltage scales). Here, the relative magnitudes of flow and no flow (i.e., simulating an occlusive event) conditions are shown.

**FIGURE 7.**
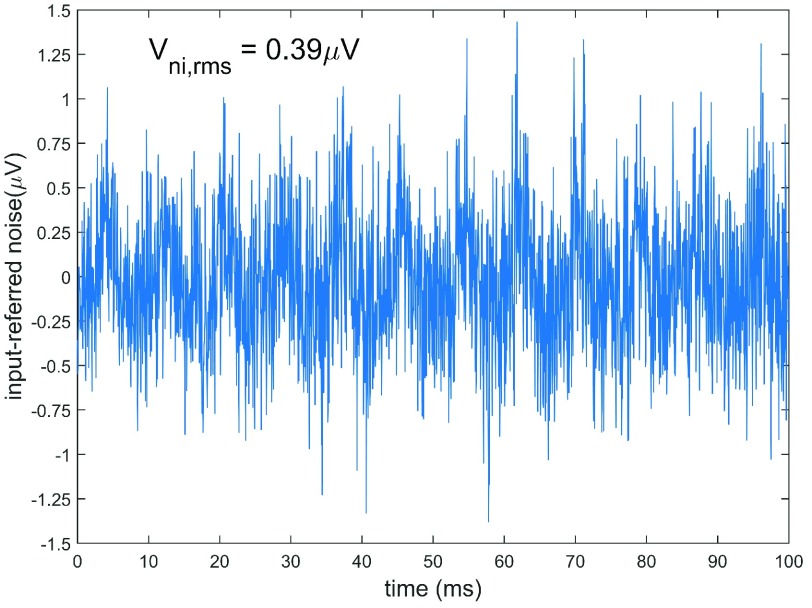
Input-referred noise voltage at the LNA input (i.e., output-referred noise divided by receiver chain gain), and its RMS value, $\text{V}_{ni,rms}$, is $0.39~\mu \text{V}$ across a 10 kHz sampled bandwidth. Common mode noise is visible at 129 Hz, which is the period between radio packet transmissions.

Figure 7 shows the input-referred voltage noise of the device, the differential voltage noise was measured at the ADC inputs and divided by the midband receiver chain voltage gain, 88 dB, referred to the LNA input. The LNA’s input is terminated with, and impedance matched to, the receiver-side piezoelectric transducer. The input-referred RMS voltage noise is $0.39~\mu \text{V}$ across a 10 kHz sampled bandwidth. Note the visible common mode noise at 129 Hz, which is a result of radio interference from the MCU’s radio, which transmits a packet every 7.74 ms. The sensitivity, defined as double the output-referred RMS noise voltage, of the receiver chain is about −113 dBm (i.e., about 20 mV$_{peak}$ at the ADC) with the LNA input matched to a $50\Omega $ vector signal generator (model #: E4438C, Agilent, Santa Clara, CA). The input dynamic range is about 35 dB, and the output voltage gain variation for an 400 Hz input tone (i.e., corresponding to about a 2.2 cm/s velocity) from −100 dBm to −78 dBm is 0.60 dB ±0.94 dB.

**FIGURE 9.**
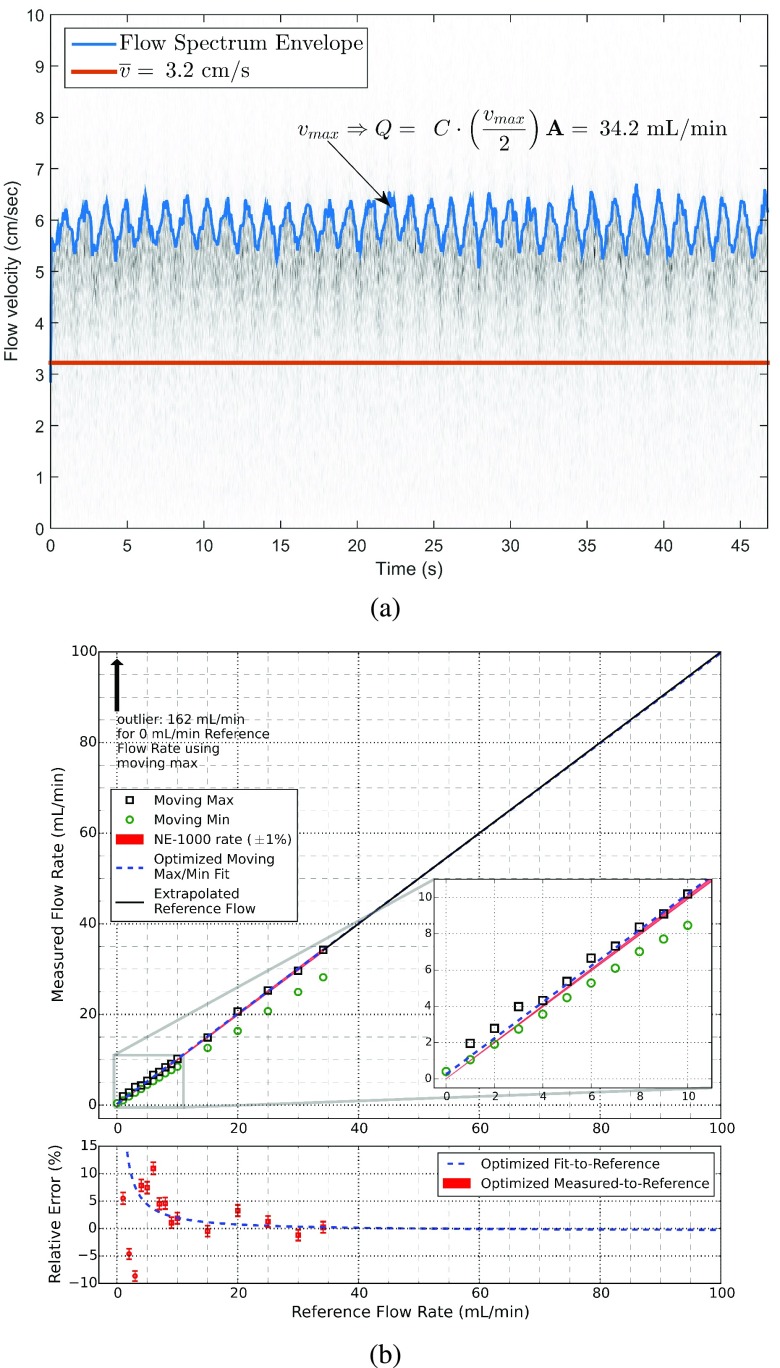
*In vitro* performance of the Doppler device using a syringe pump to generate reference flow rates. (a) Effectiveness of the moving maximum filter applied to the envelope of the time-frequency spectrogram of the Doppler signal. The reference flow rate was 34.15 mL/min, and the flow estimate was 34.2 mL/min. Note $C\,=\,60\tfrac {sec}{min}$; (b) Performance evaluation of the Doppler device comparing measured flow rate along with the corresponding magnitude of the relative error. Note that the relative error for 0.00 mL/min was omitted from the figure because its value is undefined.

Figure 9a shows the effectiveness of using a moving maximum filter to smooth the time-frequency spectrogram’s envelope of the Doppler signal. For this figure, the expected flow was 34.15 mL/min (±1%) compared to the procedure’s estimate of 34.2 mL/min, a relative error between −0.8% and +1.2%. Figure 9b shows the performance of the Doppler device across various expected flow rates, as pumped by the NE-1000 syringe pump. A moving minimum filter reduced measurement error below 4.00 mL/min flow rates, while a moving maximum filter reduced the error for rates above 3.00 mL/min. The error bars encompass the relative error range due to the ±1% NE-1000 accuracy. The large relative errors for low flow rates appears significant; however, the absolute error in this flow rate range is small. For example, at 6.00 mL/min the relative error is the worst across the entirety of measured data (i.e., between +12.1% and +9.8%), but the absolute error is between +0.60 mL/min to +0.72 mL/min. Additionally, for 0.00 mL/min, the absolute error is +0.40 mL/min; whereas, the relative error is undefined. Above 6.00 mL/min, the measured flow rate compared to the NE-1000 nameplate reference flow rate is below below ±5.0%. Additionally, when considering the error bars, for flow rates above 8.00 mL/min, the relative error is within ±5.0% of the reference flow rate, with the relative error tending towards about 0.0% as the flow rate increases. A simple linear fit to the measured data shows an absolute error that approaches about +0.24 mL/min at 0.00 mL/min and about −0.23 mL/min at the largest extrapolated flow range, 100 mL/min. The key results are that above a few mL/min in the measured range, the system will overestimate the flow rate, and for even low flow rates (i.e., above 6.00 mL/min), the relative error is small, and as the flow rate increases, the accuracy of the measurement increases as well.

### B. In Vivo Results

**FIGURE 12.**
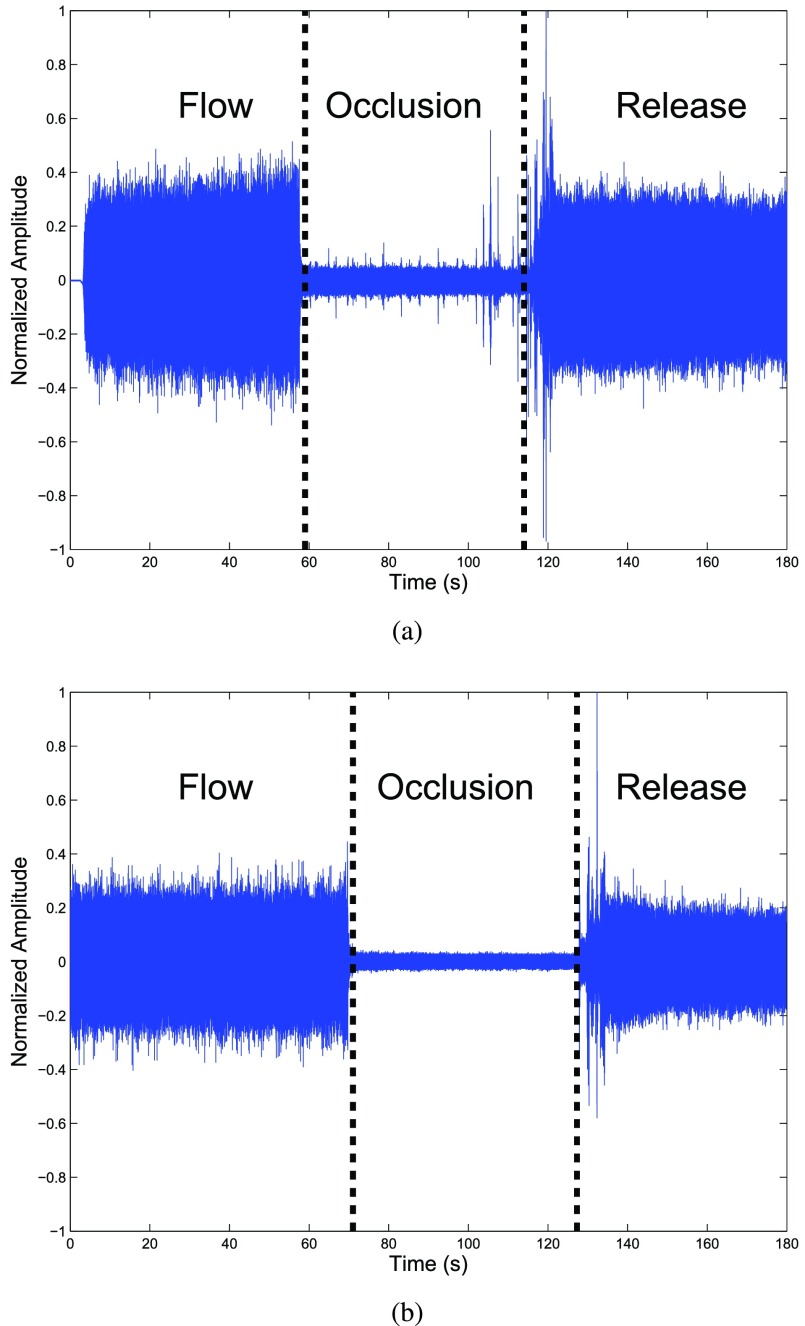
(a) Collected Doppler signal from the femoral vein in a swine’s left thigh using Device 1. (b) Collected Doppler signal from the femoral vein in the same swine’s right thigh using Device 2. Flow and no-flow/occlusion conditions are shown in the figures using the experimental data collection protocol from Section II-C.

Figure 12 shows blood flow data collected from two swine femoral veins. These results confirm the ability of the implanted Doppler device to monitor blood flow wirelessly, without the problematic tether to a bedside monitoring device. The flow and no-flow portions of the waveform in Figure 12 are displayed using the raw ADC samples (i.e., without digital signal processing). In the figure, distinctive regions of flow and no-flow are clearly visible, and the restoration of flow (i.e., “release” in the figure) after an occlusion shows the blood flow waveform approaching nearly the same amplitude as the pre-occlusion event.

**FIGURE 10.**
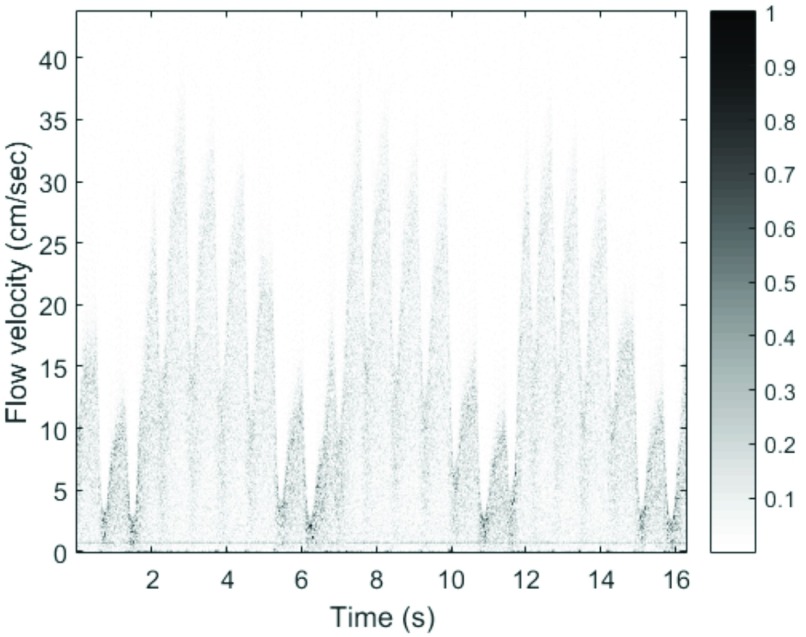
Spectrogram of blood flow across a number of cardiac cycles. Various sources of noise are visible, including modulation due to the ventilator as well as narrowband noise, due to the radio, throughout the time window.

In the figure, noise appears to be present during the no-flow portion of the waveform. Potential sources of noise can be a result of many culprits. Mixing spurs generated in-band, local oscillator phase noise, tuned circuits formed by the many bypass networks on the PCB, the boost converter, or even insufficient decoupling to prevent noise from being injected into the power supply by broadband nonlinear circuitry, such as by the mixer. Noise due to the surgeon mechanically manipulating the tourniquet around the vessel is visible near the boundary of “occlusion” and “release.” Biological noise also contributes to the noisy baseline. Blood flow in nearby adjacent vessels will be picked up by the transducers. Additionally, the pig’s respiration was controlled by a ventilator which regulated the pig’s breathing about 11–12 breaths per minute; respiration is well-known to modulate flow in the veins of the legs [68]. Figure 10 shows a spectrogram of a blood flow segment across a number of cardiac cycles. In the figure, modulation due to the ventilator is clearly shown; the start of a breath is noticeable at about 1.5 sec., 6.2 sec., and 11.6 sec. Additionally, throughout the time window, a source of narrowband noise is visible at about 0.72 cm/s (i.e., about 129 Hz), which corresponds to the period between radio packet transmissions.

**FIGURE 11.**
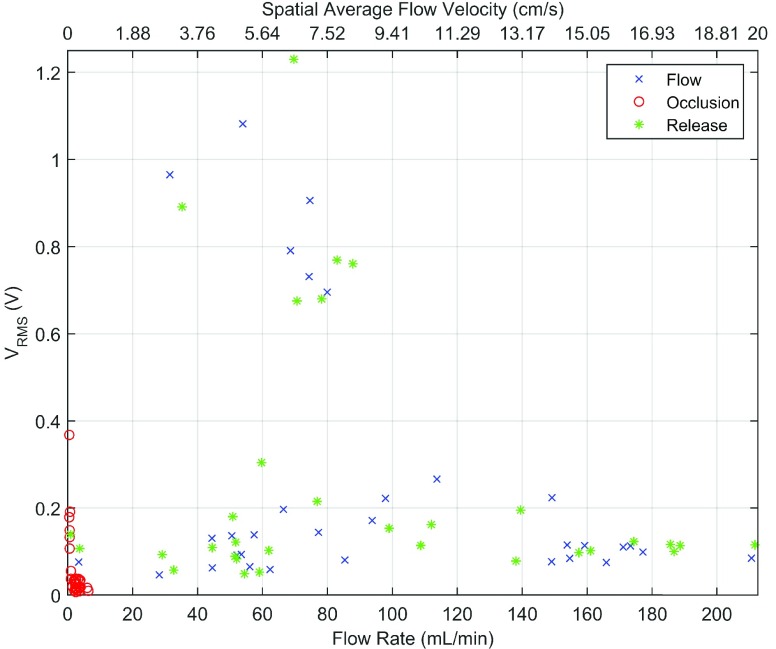
Aggregation of 32 data sets. Each data set is segmented into three groups, “flow,” “occlusion,” and “release.” The “occlusion” data points showing a larger RMS voltage value correspond to the “flow” and “release” data points clustered towards the top of the figure with correspondingly higher RMS voltage values, where all but one of these outliers come from the same pig.

Figure 11 shows an aggregate of the 32 data sets collected in this study (i.e., four sets per hind leg per pig). Using the raw blood flow waveforms (i.e., as in Figure 12), the “flow,” “occlusion,” and “release” segments of each data set were visually segmented (i.e., 96 data points in the figure). Portions of the waveform showing obvious operator involvement (i.e., particularly evident when the surgeon releases the tourniquet to resume flow) were avoided in the segmentation. The plotted data shows features for flow rate (i.e., proportional to flow velocity) and the RMS voltage of the collected time-domain raw Doppler signals. To compute the RMS voltage for a signal segment, first, a raw Doppler signal segment’s envelope is found using the optimized moving minimum/maximum filter (i.e., 0–3.00 mL/min: moving minimum, ≥ 4.00 mL/min: moving maximum filter) as used in the *in vitro* performance evaluation. Second, the RMS voltage is obtained by computing the RMS value of the envelope. It should be noted that neither the RMS voltage nor flow rate/velocity would be computed to alert a clinician to a vessel’s patency. Rather, the computed RMS voltage approximates the observed magnitude of the raw Doppler waveform’s by a clinician, and the computed flow rate/velocity, for which the calibration results were described in Section III-A, serves to confirm the true patency status of a vessel for the shown RMS voltage value. As an additional note, the “occlusion” data points showing a larger RMS voltage value correspond to the “flow” and “release” data points clustered towards the top of the figure with correspondingly higher RMS voltage values. All but one of these outliers come from the same pig.

Using the raw Doppler signal blood flow data, the average mean value and the average standard deviation were computed, for each of the three segments of the 32 data sets. Table 1 shows, expectedly, that the average mean for all three segments is about 0, and 2 to 3 orders of magnitude smaller than the average standard deviation. The average standard deviation for both the “flow” and “release” are about 3 times larger than that of the “occlusion” state, which is expected.

**TABLE 1:** Average Mean ($\mu _{avg}$) and Average Standard Deviation $(\sigma _{avg}$) for all Three Flow Segments. “*” Denotes Multiplication With $10^{-4}$

	Flow	Occlusion	Release
$\mu _{avg}$ (V)	(−2.0 ± 10)*	(−1.7 ± 10)*	(−2.0 ± 11)*
$\sigma _{avg}$ (V)	0.11 ± 0.14	0.030 ± 0.048	0.12 ± 0.18

Additionally, it is worth noting that the *in vivo* results demonstrate the developed system’s ability to arbitrate capturing blood flow from a specific device without interference from nearby devices (i.e., as pictured in Figure 6).

## IV. Discussion

The use of Doppler ultrasound to monitor blood flow is a proven tool for general physiological monitoring. Chronic implantation monitoring, postoperative monitoring, and even freely behaving subject monitoring can benefit from wireless blood flow systems. We demonstrated a tool to simplify blood flow monitoring in veins of the lower limbs, which are particularly difficult cases for ultrasound operators [56], [57].

A simple real-time display was shown. Similar to the wired gold standard in free flap monitoring, our system reports vessel patency in a simple format. The *in vitro* performance evaluation showed close agreement with the reference flow rates. Above 8.00 mL/min, its relative error is within ±5.0% for the measured data (i.e., including the instrument uncertainty of the NE-1000), and fitting to the measured data uncovers maximum absolute errors of +0.24 mL/min for a 0.00 mL/min reference and about −0.23 mL/min at the largest extrapolated flow range, 100 mL/min. Other reported continuous wave wireless totally implantable Doppler devices in literature describe their accuracy in various ways. Using a steady state flow simulator, Meindl and Di Pietro report a ±20% center velocity accuracy compared to theory, across any vessel size with any flow profile [61]. Yonezawa et al. reported ±1% linearity to the reference flow value, via a timed volume collection method, between 20 cm/s −150 cm/s flow velocities [49]. Vilkomerson et al. reported velocity accuracy errors less than 5% over a 100 mL/min to 900 mL/min range using a volume collection method [54], [69]. Using custom-built flow phantom, comprising a gear pump to hydraulically compress a pair of bellows filled with a blood-mimicking fluid [70] in an alternating fashion followed by a volume collection method, Cannata et al. reported average flow velocity estimate errors less than 6% for 30 measurements over a 2.5-hour period across a 60 mL/min to 500 mL/min flow rate range [55]. Additionally, Cannata et al reported a 1.7% lower peak flow velocity compared with a duplex ultrasound system when measuring a rabbit’s infrarenal aorta. Tang et al. described a system with about 5.83% deviation from the expected flow velocity, 24 cm/s, using a timed volume method, but only for a single reported measurement [52]. Considering the various descriptions and conditions of reported device accuracy, the calibrated accuracy of our presented system appears to report a better accuracy over its calibrated range (i.e., 0.00 mL/min to 34.15 mL/min; 0.00 cm/s to 3.2 cm/s) than found elsewhere in literature (i.e., note that the relative error, described in our calibrated results, is misleading for very low flow rates). It should be noted that the accuracy of the described system relies not only on the implanted wireless Doppler device, but the flow estimation methodology (i.e., averaging filters used in the time-frequency domain) described in Section II-C.

The *in vivo* results show that, while the majority of “occlusion” segments were clustered near 0 mL/ms with near 0 $\text{V}_{RMS}$ amplitude, there was a minority grouping that showed non-zero RMS voltages for near-zero flow rates. This minority grouping also shows concomitant larger “flow” and “release” RMS values compared to those of the rest of the data in Figure 11 (i.e., clustered near the top of the figure). The reason for the discrepancy is that flow/velocity estimation begins with a moving minimum filter, and only uses a moving maximum filter if the flow rate is greater than 3.00 mL/min; whereas, the RMS voltage estimation always uses a moving maximum filter, leading to non-zero RMS voltages for near-zero flow rates. Mechanical perturbations of the pig’s body due to a ventilator, which is used to control the pig’s respiration, manifest as periodic modulation of the raw Doppler waveform (See Figure 10). This periodic noise results in larger accumulated overestimation of the envelope when using a moving maximum filter. Therefore, data in which the periodic noise due to the ventilator is more pronounced will result in, for the “occlusion” case, as an example, larger non-zero RMS voltages while the flow rate/velocity remaining still near-zero. As a consequence, the implication is that, when using this developed Doppler system, establishing a baseline RMS voltage reading is necessary so that future assessments of anastomotic patency are valid. For example, if no baseline RMS value (i.e., baseline flow and baseline no-flow magnitudes) was established for the “occlusion” value near 0.37 V RMS, it is likely that a clinician would report a false-positive for blood flow (i.e., the flow rate for this data point is actually near 0 mL/min while the raw Doppler signal magnitude is larger than that for “flow” or “release” of other pigs) if only the absolute magnitude of the signal were considered. Nonetheless, it should be noted that in the majority data group, the absolute magnitude of the “occlusion” RMS voltage value predictably falls below the “flow” and “release” RMS voltage values.

Several outliers in the *in vivo* results (Figure 11) show a non-zero RMS voltage for a near-zero “flow” and “release” flow velocity/rate: one “flow”/“release” pair and another lone “release” component. Both can be explained by poor transducer coupling, likely due to operator involvement, resulting in a low signal-to-noise ratio and leading to a poor flow/velocity estimation. The ventilator noise resulted in the non-zero RMS voltage. The calibration procedure was void of noise and perturbations. So while the calibrated data show close agreement with theory, it is not known the degree to which noise and perturbations, such as those introduced by the pig’s ventilator, affect the estimate of flow rate.

The apparent difference in the Doppler signal voltage magnitudes in Figure 12 requires elaboration, as it is reasonable to expect the same magnitude post-occlusion as observed during pre-occlusion. Changes in blood flow after free flap transfer surgery are well-known; the reperfusion of ischemic tissue is not immediate, and resumption of flow to the pre-occlusion rate can vary widely, from minutes [71] to days [72]. The blood flow changes are due to vascular and hemodynamic responses, namely vasoconstriction (i.e., a decrease in lumen diameter) and vasodilation (i.e., an increase in lumen diameter), as a result of the reperfusion after sustained loss of flow.

Our results for the Doppler signal voltage magnitude in the “release” phase are corroborated through multiple sources. Firstly, Hjortdal et al. confirmed that the venous outflow in pig flaps is expected to increase over the first hour after the onset of perfusion [73], indicating that our “release” signal magnitude should not be expected to match the “flow” magnitude during our one minute measurement window. Second, Saltzman et al. reported brief periods of vasoconstriction (i.e., less than 10 minutes) followed by a longer period of vasodilation (i.e., peaking at 120 min.) [74] in the post-operative reperfusion period, and further concluded that these responses are dependent on the duration of the blood flow restriction. Saltzman et al.’s findings indicate that the post-occlusion vessel geometry is inhomogeneous and in flux, and thus results in a disturbed flow profile (i.e., the entrance length criteria for a well-defined flow profile is violated) – turbulence. While a turbulent flow profile is known to increase the backscattered power [75], Wang and Shung showed, in a pig animal model, that flow disturbances following the removal of an occluding ligature significantly influence the received signal magnitude, and furthermore, reported the received signal magnitude actually decreasing with increasing distance downstream, up through 3 cm [76]. The probe location in our experiments was in close proximity distally to the occlusion, and therefore, the magnitude of the Doppler signal voltage is expectedly lower than in the pre-occlusion waveform portion of Figure 12.

To the best of the authors’ knowledge, considering the electronics and antenna size, we have demonstrated the smallest wireless implantable blood flow monitor device that incorporates an MCU. The PCB area is about 8.30 cm^2^ (i.e., single side). The electronics, including the antenna and without battery, is about 1.70 cm^3^, and the total encapsulated volume, without transducer cuff and transducer leads, is about 18.0 cm^3^. Because the implant lifetime was short, encapsulation was not optimized, which added significant bulk (i.e., 6.08 cm^3^ without encapsulation; includes electronics and battery). Total implant volume can be reduced through the use of alternative encapsulation materials (e.g., silicones, epoxies, glass, etc. [77], [78]). Kiourti and Nikita provide suggestions for biocompatible encapsulation options for implantable antennas in order to minimize power loss [66]. The wireless blood flow monitor PCBs developed by Vilkomerson et al. appears to occupy about 30.5 cm^2^ (i.e., total, for both PCBs, single side) from published images [54]. The implant volume is not given. Cannata’s work stated that it used a modified version of Vilkomerson’s design, without commenting on the specific size [55]. Another device, developed by Tang, Vilkomerson, and Chilipka, demonstrated an implantable blood flow monitor with a wireless rechargeable battery; the battery was recharged by a coil [52]. The reported total PCB area (i.e., for two boards) was 7 cm^2^, but implant volume (i.e., with batteries, without batteries, without encapsulation, etc.) was not reported. Additionally, the implant volume impact by the coil was not reported. Additionally, one reported device in literature, called an anastomotic patency monitor, which used a microcontroller, reported good agreement between its measured flow and actual flow [79]; however, size and design specifics were omitted.

Previous microcontroller-equipped wireless blood flow monitors have not focused significant attention on the impact of circuit selection, performance, degree of integration, and topology to reduce PCB real-estate and implant size. Our device was able to achieve its size through the following considerations. High density PCBs which mount components on both sides and in close proximity reduce size, but interference is a significant concern, so care was taken to avoid a troublesome layout while minimizing real-estate. Differential electronics were used wherever possible to minimize the effects of coupled noise, thereby allowing for a more compact layout. Power supply decoupling was incorporated, and dedicated voltage regulators for most portions of the design were used to prevent noisy circuits from polluting power supply lines. The MCU, which incorporated both a microcontroller and a radio, saved significant real-estate by offering a high degree of functionality in a single package. The radio telemetry frequency was selected such that the antenna’s size would not incur a significant real-estate penalty. However, the caveat is that while higher frequencies typically permit smaller antenna geometries [80], the losses in biological tissues at higher frequencies are often greater [81].

The same MCU that allows software customization to control system functions, blood flow data capturing, and low power modes, can also be used to develop a scalable system, which can interrogate and control multiple devices. Examples where multiple monitors need to be used in close proximity include, free flaps requiring in-flow and out-flow monitoring, and hospitals with nearby patients being monitored. Thus far, there has been no report in literature of wireless blood flow monitoring systems supporting multiple monitors. With our developed system, a specific monitor could be activated, wirelessly, without activating unwanted nearby monitors.

The MCU software can be readily customized for specific applications. For example, the software could be customized to set the sleep timer duration dynamically, thereby extending implant lifetime and enabling chronic implantation applications. As another example, the abort key could be modified in our current implementation. Currently, all devices respond to the same abort sequence, as a failsafe to prevent accidentally leaving a device activated and draining its battery. For a large scale deployable system, this should be changed to only abort a specific device. The current system uses 16-bit abort keys and “Wake Up” SIDs. This means that $2^{16}/2$ unique devices can be multiplexed (i.e., one unique abort key and one unique “Wake Up” SID per device).

## V. Conclusion

This paper demonstrates a wireless Doppler blood flow monitoring system that reduces flow interpretation difficulties by providing a simple real-time visual indicator of blood flow. The developed system’s blood flow rate accuracy is high across its calibrated range. Careful selection of circuit performance, degree of integration, and topology resulted in a compact implant size. The microcontroller unit played a crucial role in size reduction. Additionally, it allowed implementing customized software, which extended battery life and permitted the activation of specific blood flow monitors in the presence of nearby monitors. In the future, the customizable software can be adapted to deploy a large scale monitoring system with thousands of monitors accessible from just a single external hub. And most importantly, system functions that heavily impact battery life can be dynamically set via the microcontroller unit in order to satisfy chronic implantation applications.

## References

[1] Kang M. J., Chung C. H., Chang Y. J., and Kim K. H., “Reconstruction of the lower extremity using free flaps,” Arch. Plastic Surgery, vol. 40, no. , pp. 575–583, 2013. 10.5999/aps.2013.40.5.575 PMC3785593 24086813

[2] Organek A. J., Klebuc M. J., and Zuker R. M., “Indications and outcomes of free tissue transfer to the lower extremity in children: Review,” J. Reconstruct. Microsurgery, vol. 22, no. 3, pp. 173–182, 2006. 10.1055/s-2006-939963 16780046

[3] Zhou W., He M., Liao Y., and Yao Z., “Reconstructing a complex central facial defect with a multiple-folding radial forearm flap,” J. Oral Maxillofacial Surgery, vol. 72, no. 4, pp. 836.e1–836.e4, Apr. 2014. 10.1016/j.joms.2013.12.027 24491846

[4] Yanko-Arzi R. et al., “The role of free tissue transfer in posterior neck reconstruction,” J. Reconstruct. Microsurgery, vol. 30, no. 5, pp. 305–312, 2014. 10.1055/s-0033-1361841 24399697

[5] Rosson G. D. et al., “A review of the surgical management of breast cancer: Plastic reconstructive techniques and timing implications,” Ann. Surgical Oncol., vol. 17, no. 7, pp. 1890–1900, Jul. 2010. 10.1245/s10434-010-0913-7 20217253

[6] Urken M. L. et al., “Microvascular free flaps in head and neck reconstruction: Report of 200 cases and review of complications,” Arch. Otolaryngol.–Head Neck Surgery, vol. 120, no. 6, pp. 633–640, 1994. 10.1001/archotol.1994.01880300047007 8198786

[7] Tiwari V. K., Sarabahi S., and Chauhan S., “Preputial flap as an adjunct to groin flap for the coverage of electrical burns in the hand,” Burns, vol. 40, no. 1, pp. e4–e7, 2014. 24035578 10.1016/j.burns.2013.06.017

[8] Firat C. and Geyik Y., “Surgical modalities in gunshot wounds of the face,” J. Craniofacial Surgery, vol. 24, no. 4, pp. 1322–1326, Jun. 2013. 10.1097/SCS.0b013e31829978c3 23851799

[9] Papel I. D. et al., Facial Plastic and Reconstructive Surgery, 3rd ed. New York, NY, USA: Thieme, 2009.

[10] Taylor G. I. and Daniel R. K., “The anatomy of several free flap donor sites,” Plastic Reconstruct. Surgery, vol. 56, no. 3, pp. 243–253, 1975. 10.1097/00006534-197509000-00001 1098069

[11] Attinger C. E., Ducic I., Hess C. L., Basil A., Abbruzzesse M., and Cooper P., “Outcome of skin graft versus flap surgery in the salvage of the exposed Achilles tendon in diabetics versus nondiabetics,” Plastic Reconstruct. Surgery, vol. 117, no. 7, pp. 2460–2467, 2006. 10.1097/01.prs.0000219345.73727.f5 16772957

[12] Godina M., “Preferential use of end-to-side arterial anastomoses in free flap transfers,” Plastic Reconstruct. Surgery, vol. 64, no. 5, pp. 673–682, 1979. 388482

[13] O’Brien B. M., Morrison W. A., Ishida H., MacLeod A. M., and Gilbert A., “Free flap transfers with microvascular anastomoses,” Brit. J. Plastic Surgery, vol. 27, no. 3, pp. 220–230, Jul. 1974. 4607396

[14] Goodstein W. A. and Buncke H. J. Jr., “Patterns of vascular anastomoses vs. success of free groin flap transfers,” Plastic Reconstruct. Surgery, vol. 64, no. 1, pp. 37–40, 1979. 10.1097/00006534-197907000-00007 377330

[15] Strauch B. and Yu H.-L., Atlas of Microvascular Surgery: Anatomy and Operative Techniques. New York, NY, USA: Thieme, 2006.

[16] Swartz W. M. and Banis J. C., Head and Neck Microsurgery. Baltimore, MD, USA: Williams & Wilkins, 1992.

[17] Paydar K. Z., Hansen S. L., Chang D. S., Hoffman W. Y., and Leon P., “Implantable venous Doppler monitoring in head and neck free flap reconstruction increases the salvage rate,” Plastic Reconstruct. Surgery, vol. 125, no. 4, pp. 1129–1134, 2010. 10.1097/PRS.0b013e3181d0ab23 20335864

[18] Wu C.-C., Lin P.-Y., Chew K.-Y., and Kuo Y.-R., “Free tissue transfers in head and neck reconstruction: Complications, outcomes and strategies for management of flap failure: Analysis of 2019 flaps in single institute,” Microsurgery, vol. 34, no. 5, pp. 339–344, 2014. 24318866 10.1002/micr.22212

[19] Chen K.-T. et al., “Timing of presentation of the first signs of vascular compromise dictates the salvage outcome of free flap transfers,” Plastic Reconstruct. Surgery, vol. 120, no. 1, pp. 187–195, 2007. 10.1097/01.prs.0000264077.07779.50 17572562

[20] Novakovic D., Patel R. S., Goldstein D. P., and Gullane P. J., “Salvage of failed free flaps used in head and neck reconstruction,” Head Neck Oncol., vol. 1, no. 1, pp. 1–5, 2009. 19698095 10.1186/1758-3284-1-33 PMC2749848

[21] Fischer J. P. et al., “Free tissue transfer in the obese patient: An outcome and cost analysis in 1258 consecutive abdominally based reconstructions,” Plastic Reconstruct. Surgery, vol. 131, no. 5, pp. 681e–692e, 2013. 10.1097/PRS.0b013e31828e2159 23629107

[22] Ling X. F. and Peng X., “What is the price to pay for a free fibula flap? A systematic review of donor-site morbidity following free fibula flap surgery,” Plastic Reconstruct. Surgery, vol. 129, no. 3, pp. 657–674, 2012. 10.1097/PRS.0b013e3182402d9a 22090247

[23] Momoh A. O., Yu P., Skoracki R. J., Liu S., Feng L., and Hanasono M. M., “A prospective cohort study of fibula free flap donor-site morbidity in 157 consecutive patients,” Plastic Reconstruct. Surgery, vol. 128, no. 3, pp. 714–720, 2011. 10.1097/PRS.0b013e318221dc2a 21572380

[24] Shestak K. C. and Jones N. F., “Microsurgical free-tissue transfer in the elderly patient,” Plastic Reconstruct. Surgery, vol. 88, no. 2, pp. 259–263, 1991. 10.1097/00006534-199108000-00014 1852818

[25] Ferguson R. E. Jr., and Yu P., “Techniques of monitoring buried fasciocutaneous free flaps,” Plastic Reconstruct. Surgery, vol. 123, no. 2, pp. 525–532, 2009. 10.1097/PRS.0b013e318196b9a3 19182610

[26] Smit J. M., Whitaker I. S., Liss A. G., Audolfsson T., Kildal M., and Acosta R., “Post operative monitoring of microvascular breast reconstructions using the implantable Cook–Swartz Doppler system: A study of 145 probes & technical discussion,” J. Plastic, Reconstruct. Aesthetic Surgery, vol. 62, no. 10, pp. 1286–1292, Oct. 2009. 10.1016/j.bjps.2008.06.007 18675608

[27] Cho B. C., Shin D. P., Byun J. S., Park J. W., and Baik B. S., “Monitoring flap for buried free tissue transfer: Its importance and reliability,” Plastic Reconstruct. Surgery, vol. 110, no. 5, pp. 1249–1258, 2002. 10.1097/01.PRS.0000025286.03909.72 12360063

[28] Fischer J. P. et al., “Comprehensive outcome and cost analysis of free tissue transfer for breast reconstruction: An experience with 1303 flaps,” Plastic Reconstruct. Surgery, vol. 131, no. 2, pp. 195–203, 2013. 10.1097/PRS.0b013e318277856f 23357982

[29] Kind G. M., Buntic R. F., Buncke G. M., Cooper T. M., Siko P. P., and Buncke H. J. Jr., “The effect of an implantable Doppler probe on the salvage of microvascular tissue transplants,” Plastic Reconstruct. Surgery, vol. 101, no. 5, pp. 1268–1273, 1998. 9529212

[30] Swartz W. M., Izquierdo R., and Miller M. J., “Implantable venous Doppler microvascular monitoring: Laboratory investigation and clinical results,” Plastic Reconstruct. Surgery, vol. 93, no. 1, pp. 152–163, 1994. 10.1097/00006534-199401000-00024 8278470

[31] Holm C., Tegeler J., Mayr M., Becker A., Pfeiffer U. J., and Mühlbauer W., “Monitoring free flaps using laser-induced fluorescence of indocyanine green: A preliminary experience,” Microsurgery, vol. 22, no. 7, pp. 278–287, 2002. 12404345 10.1002/micr.10052

[32] Irwin M. S., Thorniley M. S., Doré C. J., and Green C. J., “Near infra-red spectroscopy: A non-invasive monitor of perfusion and oxygenation within the microcirculation of limbs and flaps,” Brit. J. Plastic Surgery, vol. 48, no. 1, pp. 14–22, 1995. 10.1016/0007-1226(95)90024-1 7719602

[33] Keller A., “Noninvasive tissue oximetry for flap monitoring: An initial study,” J. Reconstruct. Microsurgery, vol. 23, no. 4, pp. 189–198, 2007. 10.1055/s-2007-974655 17530610

[34] Khouri R. K. and Shaw W. W., “Monitoring of free flaps with surface-temperature recordings: Is it reliable?” Plastic Reconstruct. Surgery, vol. 89, no. 3, pp. 495–499, 1992. 1741473

[35] Stone C. A., Dubbins P. A., and Morris R. J., “Use of colour duplex Doppler imaging in the postoperative assessment of buried free flaps,” Microsurgery, vol. 21, no. 5, pp. 223–227, 2001. 11494397 10.1002/micr.1043

[36] Swartz W. M., Jones N. F., Cherup L., and Klein A., “Direct monitoring of microvascular anastomoses with the 20-MHz ultrasonic Doppler probe: An experimental and clinical study,” Plastic Reconstruct. Surgery, vol. 81, no. 2, pp. 149–158, 1988. 10.1097/00006534-198802000-00001 3336646

[37] Fernando B., Young V. L., and Logan S. E., “Miniature implantable laser Doppler probe monitoring of free tissue transfer,” Ann. Plastic Surgery, vol. 20, no. 5, pp. 434–442, 1988. 10.1097/00000637-198805000-00006 2967664

[38] Parker P. M., Fischer J. C., and Shaw W. W., “Implantable pulsed Doppler cuff for long-term monitoring of free flaps: A preliminary study,” Microsurgery, vol. 5, no. 3, pp. 130–135, 1984. 6493028 10.1002/micr.1920050307

[39] Smit J. M., Zeebregts C. J., Acosta R., and Werker P. M., “Advancements in free flap monitoring in the last decade: A critical review,” Plastic Reconstruct. Surgery, vol. 125, no. 1, pp. 177–185, 2010. 10.1097/PRS.0b013e3181c49580 20048610

[40] Guillemaud J. P., Seikaly H., Cote D., Allen H., and Harris J. R., “The implantable Cook–Swartz Doppler probe for postoperative monitoring in head and neck free flap reconstruction,” Arch. Otolaryngol.–Head Neck Surgery, vol. 134, no. 7, pp. 729–734, 2008. 10.1001/archotol.134.7.729 18645123

[41] Pryor S. G., Moore E. J., and Kasperbauer J. L., “Implantable Doppler flow system: Experience with 24 microvascular free-flap operations,” Otolaryngol.-Head Neck Surgery, vol. 135, no. 5, pp. 714–718, 2006. 10.1016/j.otohns.2006.05.006 17071300

[42] Oliver D. W., Whitaker I. S., Giele H., Critchley P., and Cassell O., “The Cook–Swartz venous Doppler probe for the post-operative monitoring of free tissue transfers in the United Kingdom: A preliminary report,” Brit. J. Plastic Surgery, vol. 58, no. 3, pp. 366–370, Apr. 2005. 10.1016/j.bjps.2004.12.003 15780232

[43] Rozen W. M. et al., “Sutured attachment of the implantable Doppler probe cuff for large or complex pedicles in free tissue transfer,” J. Reconstruct. Microsurgery, vol. 27, no. 2, pp. 99–102, 2011. 10.1055/s-0030-1267837 20945281

[44] Nelson T. R. and Pretorius D. H., “The Doppler signal: Where does it come from and what does it mean?” Amer. J. Roentgenol., vol. 151, no. 3, pp. 439–447, 1988. 2970215 10.2214/ajr.151.3.439

[45] Law Y. F., Bascom P. A., Johnston K. W., Vaitkus P., and Cobbold R. S. C., “Experimental study of the effects of pulsed Doppler sample volume size and position on the Doppler spectrum,” Ultrasonics, vol. 29, no. 5, pp. 404–410, Sep. 1991. 1882486 10.1016/0041-624x(91)90093-n

[46] Shung K. K. and Thieme G. A., Ultrasonic Scattering in Biological Tissues. Boca Raton, FL, USA: CRC Press, 1992.

[47] Rosenberg J. J., Fornage B. D., and Chevray P. M., “Monitoring buried free flaps: Limitations of the implantable Doppler and use of color duplex sonography as a confirmatory test,” Plastic Reconstruct. Surgery, vol. 118, no. 1, pp. 109–113, 2006. 10.1097/01.prs.0000221113.78244.8c 16816680

[48] Franklin D. E., Watson N. W., Pierson K. E., and Van Citters R. L., “Technique for radio telemetry of blood-flow velocity from unrestrained animals,” Amer. J. Med. Electron., vol. 5, no. 1, pp. 24–28, 1965. 4956018

[49] Yonezawa Y., Nakayama T., Ninomiya I., and Caldwell W. M., “Radio telemetry directional ultrasonic blood flowmeter for use with unrestrained animals,” Med. Biol. Eng. Comput., vol. 30, no. 6, pp. 659–665, Nov. 1992. 1297024 10.1007/BF02446801

[50] Gill R. W. and Meindl J. D., “Low power integrated circuits for an implantable pulsed Doppler ultrasonic blood flowmeter,” IEEE J. Solid-State Circuits, vol. 10, no. 6, pp. 464–471, Dec. 1975.

[51] DiPietro D. M. and Meindl J. D., “Integrated circuits for an implantable CW Doppler ultrasonic flowmeter,” IEEE J. Solid-State Circuits, vol. 12, no. 5, pp. 573–576, Oct. 1977.

[52] Tang S. C., Vilkomerson D., and Chilipka T., “Magnetically-powered implantable Doppler blood flow meter,” in Proc. IEEE Int. Ultrason. Symp., Sep. 2014, pp. 1622–1625.

[53] Sejdic E., Rothfuss M. A., Gimbel M. L., and Mickle M. H., “Comparative analysis of compressive sensing approaches for recovery of missing samples in implantable wireless Doppler device,” IET Signal Process., vol. 8, no. 3, pp. 230–238, 5 2014.

[54] Vilkomerson D. et al., “Implantable ultrasound devices,” Proc. SPIE, vol. 6920, pp. 69200C-1–69200C-11, Mar. 2008.

[55] Cannata J. M. et al., “Development of a flexible implantable sensor for postoperative monitoring of blood flow,” J. Ultrasound Med., vol. 31, no. 11, pp. 1795–1802, Nov. 2012. 23091251 10.7863/jum.2012.31.11.1795 PMC3762578

[56] Galeandro A. I. et al., “Doppler ultrasound venous mapping of the lower limbs,” Vascular Health Risk Manage., vol. 8, pp. 59–64, Feb. 2012. 10.2147/VHRM.S27552 PMC3282606 22371652

[57] Porter J. M., Moneta G. L., and An International Consensus Committee on Chronic Venous Disease, “Reporting standards in venous disease: An update,” J. Vascular Surgery, vol. 21, no. 4, pp. 635–645, Apr. 1995. 10.1016/s0741-5214(95)70195-8 7707568

[58] Boote E. J., “AAPM/RSNA physics tutorial for residents: Topics in US: Doppler US techniques: Concepts of blood flow detection and flow dynamics 1,” Radiographics, vol. 23, no. 5, pp. 1315–1327, 2003. 12975518 10.1148/rg.235035080

[59] Gill R. W., “Measurement of blood flow by ultrasound: Accuracy and sources of error,” Ultrasound Med. Biol., vol. 11, no. 4, pp. 625–641, Jul-Aug 1985. 2931884 10.1016/0301-5629(85)90035-3

[60] Thiriet M., Biology and Mechanics of Blood Flows: Part II: Mechanics and Medical Aspects, vol. 2 New York, NY, USA: Springer, 2007.

[61] Di Pietro D. M. and Meindl J. D., “Optimal system design for an implantable CW Doppler ultrasonic flowmeter,” IEEE Trans. Biomed. Eng., vol. BME-25, no. 3, pp. 255–264, 5 1978. 150398 10.1109/TBME.1978.326330

[62] Fogel M. A., Ventricular Function and Blood Flow in Congenital Heart Disease. New York, NY, USA: Wiley, 2008.

[63] Luo W., Hosseini H., Zderic V., Mann F., O’Keefe G., and Vaezy S., “Detection and localization of peripheral vascular bleeding using Doppler ultrasound,” J. Emergency Med., vol. 41, no. 1, pp. 64–73, Jul. 2011. 10.1016/j.jemermed.2010.01.001 20189743

[64] Park H. et al., “A wireless magnetoresistive sensing system for an intraoral tongue-computer interface,” IEEE Trans. Biomed. Circuits Syst., vol. 6, no. 6, pp. 571–585, Dec. 2012. 23853258 10.1109/TBCAS.2012.2227962 PMC4445236

[65] Kiourti A., Christopoulou M., and Nikita K. S., “Performance of a novel miniature antenna implanted in the human head for wireless biotelemetry,” in Proc. IEEE Int. Symp. Antennas Propag., Jul. 2011, pp. 392–395.

[66] Kiourti A. and Nikita K. S., “A review of implantable patch antennas for biomedical telemetry: Challenges and solutions [wireless corner],” IEEE Antennas Propag. Mag., vol. 54, no. 3, pp. 210–228, Jun. 2012.

[67] Kim J. and Rahmat-Samii Y., “Implanted antennas inside a human body: Simulations, designs, and characterizations,” IEEE Trans. Microw. Theory Techn., vol. 52, no. 8, pp. 1934–1943, Aug. 2004.

[68] Brant W. E., The Core Curriculum: Ultrasound. Philadelphia, PA, USA: Lippincott Williams & Wilkins Philadelphia, 2001.

[69] Vilkomerson D. and Chilipka T., “Implantable Doppler system for self-monitoring vascular grafts,” in Proc. IEEE Ultrason. Symp., vol. 1 Aug. 2004, pp. 461–465.

[70] Ramnarine K. V., Nassiri D. K., Hoskins P. R., and Lubbers J., “Validation of a new blood-mimicking fluid for use in Doppler flow test objects,” Ultrasound Med. Biol., vol. 24, no. 3, pp. 451–459, Mar. 1998. 9587999 10.1016/s0301-5629(97)00277-9

[71] Walkinshaw M., Engrav L., Gottlieb J., and Holloway G. A., “Flow recovery and vasoconstriction following microvascular anastomosis,” Ann. Plastic Surgery, vol. 20, no. 6, pp. 533–539, Jun. 1988. 10.1097/00000637-198806000-00006 2968773

[72] Ichinose A., Tahara S., Terashi H., Nomura T., and Omori M., “Short-term postoperative flow changes after free radial forearm flap transfer: Possible cause of vascular occlusion,” Ann. Plastic Surgery, vol. 50, no. 2, pp. 160–164, Feb. 2003. 10.1097/01.SAP.0000037264.92535.AC 12567053

[73] Hjortdal V. E., Hansen E. S., Kjølseth D., Henriksen T. B., Gottrup F., and Djurhuus J. C., “Arteriovenous shunting and regional blood flow in myocutaneous island flaps: An experimental study in pigs,” Plastic Reconstruct. Surgery, vol. 87, no. 2, pp. 326–334, 1991. 10.1097/00006534-199102000-00015 1989025

[74] Saltzman D. J. et al., “Microvascular changes following four-hour single arteriole occlusion,” Microsurgery, vol. 33, no. 3, pp. 207–215, Mar. 2013. 23152084 10.1002/micr.22051

[75] Shung K. K., Cloutier G., and Lim C. C., “The effects of hematocrit, shear rate, and turbulence on ultrasonic Doppler spectrum from blood,” IEEE Trans. Biomed. Eng., vol. 39, no. 5, pp. 462–469, 5 1992. 1526637 10.1109/10.135540

[76] Wang S.-H. and Shung K. K., “*In vivo* measurements of ultrasonic backscattering in blood,” IEEE Trans. Ultrason., Ferroelect., Freq. Control, vol. 48, no. 2, pp. 425–431, Mar. 2001. 10.1109/58.911725 11370356

[77] Zhou D. and Greenbaum E., Implantable Neural Prostheses 2: Techniques and Engineering Approaches (Biological and Medical Physics, Biomedical Engineering). New York, NY, USA: Springer, 2010.

[78] Park S., Borton D. A., Kang M., Nurmikko A. V., and Song Y.-K., “An implantable neural sensing microsystem with fiber-optic data transmission and power delivery,” Sensors, vol. 13, no. 5, pp. 6014–6031, 2013. 23666130 10.3390/s130506014 PMC3690043

[79] Read P. F. K., Martin R. W., Albrecht E. H., and Proctor A. H., “Implantable Doppler ultrasonic vessel patency monitor,” in Proc. Annu. Int. Conf. IEEE Eng. Med. Biol. Soc., Nov. 1989, pp. 1106–1107.

[80] Balanis C. A., Antenna Theory and Design. New York, NY, USA: Wiley, 2012.

[81] Gabriel S., Lau R. W., and Gabriel C., “The dielectric properties of biological tissues: II. Measurements in the frequency range 10 Hz to 20 GHz,” Phys. Med. Biol., vol. 41, no. 11, p. 2251, 1996. 8938025 10.1088/0031-9155/41/11/002

